# Prevalence of Carotid Stenosis and Incidence of Ischemic Stroke in Patients Undergoing Non-Coronary Cardiac Surgery

**DOI:** 10.21470/1678-9741-2018-0127

**Published:** 2019

**Authors:** Mario Augusto Cray da Costa, João Paulo Nadal, Jefferson Matsuiti Okamoto, André Luis Betero, Marcelo Derbli Schafranski, Ricardo Zanetti Gomes, Elise Souza dos Santos Reis

**Affiliations:** 1 Universidade Estadual de Ponta Grossa, Departamento de Medicina, Campus Uvaranas Medicina, Ponta Grossa, PR, Brazil.

**Keywords:** Stroke, Cardiac Surgical Procedures, Carotid Stenosis, Valvar Disease

## Abstract

**Introduction:**

Many publications on coronary surgery and carotid stenosis (CS) can be found, but we do not have enough information about the relationship between ischemic stroke, CS and non-coronary cardiac surgery.

**Objectives:**

To evaluate the incidence and risk factors associated with the stroke and CS ≥50% in patients undergoing non-coronary surgeries.

**Objectives:**

We assessed 241 patients, aged 40 years or older, between 2009 and 2016, operated in Santa Casa de Misericórdia de Ponta Grossa-PR, Brazil. We perform carotid Doppler in patients 40 years of age or older before any cardiac surgery as a routine. The incidence and possible risk factors for CS ≥50% and perioperative stroke were analyzed by univariate statistical analysis.

**Results:**

11 patients (4.56%) presented perioperative stroke. The risk factor for stroke was CS ≥50%: OR=5.3750 (1.2909-22.3805), *P*=0.0208. Eighteen patients (7.46%) had CS ≥50% and their risk factors were extracardiac arteriopathy: OR=18.6607 (6.3644-54.7143), *P*<0.0001; COPD: OR=3.9040 (1.4491-10.5179), *P*=0.0071; diabetes mellitus: OR=2.9844 (1.0453-8.5204), *P*=0.0411; recent myocardial infarction: OR=13.8125 (1.8239-104.6052), *P*=0.0110; EuroSCORE II higher *P*=0.0056.

**Conclusion:**

The incidences of stroke and CS ≥50% were 4.56% and 7.46%, respectively. The risk factor for stroke was CS ≥50% and for CS ≥50% were extracardiac arteriopathy, COPD, diabetes mellitus, recent myocardial infarction and higher EuroSCORE II.

**Table:** 

Abbreviations, acronyms & symbols	
CPB	= Cardiopulmonary bypass
CKD	= Chronic kidney disease
COPD	= Chronic obstructive pulmonary disease
CS	= Carotid stenosis
CT	= Cranial tomography
ECST	= European Carotid Surgery Trial
NASCET	= North American Symptomatic Carotid Endarterectomy Trial
TIA	= Transient ischemic attack

## INTRODUCTION

Many studies have correlated carotid stenosis (CS) with coronary artery disease, coronary artery bypass grafting, and stroke risk^[1-12]^. Nevertheless, the literature is poor regarding the correlation between CS and non-coronary cardiac surgery, which may result from lack of studies in the area rather than actual negative evidence of association.

Stroke is a complication that occurs in 2% of cardiac surgeries and remains one of the most important causes of mortality, morbidity and increased health expenses^[2,3]^. The incidence of stroke after valve surgery varies from 2 to 17%^[13-16]^. Risk factors for ischemic stroke in coronary surgery are: age >70 years old, female gender, systemic arterial hypertension, diabetes mellitus, chronic kidney disease (CKD), smoking, chronic obstructive pulmonary disease (COPD), peripheral arteriopathy, left ventricular ejection fraction below 40%, previous stroke or transient ischemic attack (TIA), carotid stenosis, calcified aorta, prolonged cardiopulmonary bypass (CPB), and emergency surgery, among others^[10]^. The approach of patients with carotid stenosis remains controversial^[1]^. The 2011 Guideline of the American Heart Association recommends special care for patients with severe carotid stenosis, cardiac surgery candidates, even if asymptomatic. That guideline recommends performing prophylactic treatment with endarterectomy or stenting in the carotid artery in selected cases^[11]^. However, Revelo et al.^[12]^ when reporting a Brazilian case series, demonstrated that patients with severe CS who underwent intervention for treatment during the perioperative period had worse prognosis^[12]^. On the other hand, for Naylor and Bown^[2]^ carotid lesions would be more significant markers of stroke than associated with its etiology, since these authors observed that groups of patients with unilateral stenosis above 50 or 70% did not present alterations in the incidence of stroke^[2]^. The main objective of this article is to demonstrate the correlation between CS and stroke in non-coronary cardiac surgery, with the aim of evaluating: (a) the incidence of stroke in non-coronary surgery; (b) the incidence of CS ≥50% in these patients; (c) risk factors associated with the occurrence of stroke; and (d) the risk factors associated with CS ≥50% in patients undergoing non-coronary cardiac surgery.

## METHODS

## Type of Study

After approval by the Human Research Ethics Committee of Universidade Estadual de Ponta Grossa – Paraná, Brazil, report 172,980, we developed a retrospective, observational, analytical, case-control study with information collected from a database developed prospectively at the Cardiac Surgery Service of Santa Casa de Misericórdia Hospital in Ponta Grossa – Paraná, Brazil.

## Sample Studied

We evaluated data from 270 patients aged ≥ 40 years, who underwent non-coronary surgery at Santa Casa de Misericórdia de Ponta Grossa between 2009 and 2016. We routinely perform carotid Doppler ultrasonography in all patients aged ≥ 40 years, except in urgency and emergency surgery when there is insufficient time for examination. We excluded 29 patients with incomplete information (especially lack of carotid Doppler), resulting in a sample of 241 patients.

## Endpoints

We evaluated two outcomes: stroke and presence of plaque in common or internal carotid arteries with stenosis ≥50%. Those patients who did not present any of the observed outcomes comprised the control groups of each proposed analysis.

## Data Collection

The data were obtained from a database that had been built prospectively, forming the database of the Cardiac Surgery Service of Santa Casa in Ponta Grossa with preoperative, transoperative and postoperative information, which allowed us to compose the database of EuroSCORE II^[17]^ along with other variables related to neurological complications, the focus of this work. Although the study was retrospective, the database has been developed prospectively and systematically, including all EuroSCORE II data, carotid Doppler results, cardiopulmonary bypass and aortic cross-clamping times, and presence of severe complications, including stroke.

## Studied Variables

Variables endpoints: (a) presence of perioperative stroke; (b) presence of carotid stenosis ≥50% in common or internal carotid arteries. The "perioperative" period covers the three periods of an operation: "preoperative", "transoperative" and "postoperative", and can also be defined as the period between surgical indication and return to the patient's activities. We considered strokes from the time of anesthesia to hospital discharge, since preoperative stroke was unusual. The investigation of stroke was carried out through daily evaluation of the electronic medical record by the researchers, and we considered as a stroke the presence of clinical symptoms lasting more than 1 hour compatible with ischemic injury associated with change in cranial tomography (CT) as adopted by other authors. Symptoms could be present in recovery from anesthesia, indicating a transoperative event; or in the first hours postoperatively or later, when the patient recovers from the anesthetic effect and then presents neurological alterations, characterizing a postoperative event^[18]^. CT scan of the skull is performed immediately after the onset of symptoms to rule out a hemorrhagic event and repeated at 24 and 48 hours routinely, or repeated any time, in case of a change in the neurological status. As risk factors for stroke, we analyzed the variables that make up the EuroSCORE II and some additional ones:

(a) Age (divided into <60 and ≥60 years);
(b) Gender;
(c) Renal function divided into two groups: one group with creatinine clearance ≥85 mL/min and another group with creatinine clearance <85 mL/min;
(d) Carotid stenosis ≥50%: the presence of carotid lesion was studied separately from other peripheral vascular lesions, being considered an isolated variable, unlike the EuroSCORE II, where these variables are grouped. Carotid exams are classified from normal to different degrees of obstruction, according to the European Carotid Surgery Trial method, which uses the Doppler to perform the measurements^[19]^. For the statistical calculation of the present sample, two groups of patients were considered: without stenosis or lumen obstruction up to 49%, and stenosis with an obstruction of 50% or more in the internal or common carotid artery on at least one side^[9]^. Patients with obstruction of 50% or more had their mean arterial blood pressure maintained above 70 mmHg in the perioperative period;
(e) Extracardiac arteriopathy (when the patient presents with claudication, amputation due to arterial disease in the lower limbs and previous or planned intervention in the abdominal aorta or lower limb arteries);
(f) Mobility severely compromised by neurological or muscular disease;
(g) Previous cardiac surgery;
(h) Chronic obstructive pulmonary disease;
(i) Critical preoperative state: ventricular tachycardia or ventricular fibrillation or aborted sudden death, preoperative cardiac massage, mechanical ventilation before entering the operating room, use of inotropic drugs prior to anesthesia or use of intra-aortic balloon, acute kidney failure (anuria or oliguria <10 mL/h);
(j) Diabetes mellitus;
(k) Use of insulin;
(l) NYHA functional class divided into classes I and II against classes III and IV;
(m) Left ventricular function considered normal (ejection fraction ≥50%) or altered (ejection fraction <50%). Left ventricular (LV) function was assessed by echocardiogram, considering the ejection fraction calculated by Teicholz, when there was no segmental dysfunction and by the Simpson method in the presence of segmental dysfunction or in the major alteration of ventricular geometry;
(n) Recent myocardial infarction (MI) within 90 days;
(o) Pulmonary hypertension (pulmonary systolic artery pressure >30 mmHg);
(p) Elective or urgency/emergency procedure;
(q) Type of surgical procedure. We performed operations such as aortic and mitral valve replacement or repair, interatrial or interventricular communication correction, or combined procedures;
(r) Risk by EuroSCORE II: low (up to 2); moderate (2 to 4); high (4 to 6) and very high (>6);
(s) Cardiopulmonary bypass (CPB) time.

These items were also considered risk factors for carotid stenosis, except for carotid stenosis itself and CBP time.

## Statistical Analysis

The continuous variables were presented in mean and standard deviation and the nominal variables in absolute number and percentage, the distributions were tested for normality. Student's t-test was used to compare means, and Fisher's test or chi-square test with Yates correction and logistic regression was used for the comparison of categorical variables. Values of *P*<0.05 were considered significant.

## RESULTS

In the analyzed series of 241 patients, the mean age was 59.37 years old, and 43.98% (n=106) were under 60 years old. The sample consisted of 49.37% (n=119) men, out of which 4.56% (n=11) presented perioperative stroke and 7.46% (n=18) had CS ≥50%. These and other characteristics of the sample are presented in Table 1. All cases of stroke (n=11) were ischemic, with 27.27% (n=3) in the right brain hemisphere, 63.64% (n=7) on the left, and 9.09% (n=1) bilateral. The mean age of patients with stroke was 61.19 years, 54.5% were men (n=6) and 27.3% (n=3) had carotid stenosis ≥50% (Table 2). All stroke cases portrayed in these patients were observed as ipsilateral to carotid lesions ≥50%. The surgical subtype with the highest incidence of stroke was aortic valve replacement with 63.63% of cases of stroke, while mitral surgeries accounted for 27.27% of all cases of stroke, but with no statistical difference. The incidence of stroke was 6.4% and 4.2% for aortic and mitral valve surgeries, respectively. The risk factors for stroke were compared between groups and are shown in Table 2. Among the factors evaluated, presence of carotid stenosis ≥50% was the only one presenting a statistically significant difference (Fisher's test = 0.039, OR 5.37, 95% CI 1.29-22.3, *P*=0.02) (Table 2). Considering patients with CS ≥50%, half (n=9) were men, one third (n=6) had diabetes mellitus, 61.1% (n=11) had COPD, 55.6% (n=10) had concomitant extracardiac arteriopathy, 11.1% (n=2) had recent acute myocardial infarction (Table 3). The risk factors for the presence of CS ≥50% can be observed in Table 3, in which the statistically significant ones were: extracardiac arteriopathy: OR=18.6607 (6.3644-54.7143), *P*<0.0001; COPD: OR=3.9040 (1.4491-10.5179), *P*=0.0071; diabetes mellitus: OR=2.9844 (1.0453-8.5204), *P*=0.0411; recent MI: OR=13.8125 (1.8239-104.6052), *P*=0.0110. Higher EuroSCORE II was also associated with a higher incidence of CS ≥50%, which was 2.5% in EuroSCORE II < 2, 8.5% when between 2 and 4 and 20.8% when > 4.

**Table 1:** Sample characteristics.

		Patients (n=241) n (%)
Age	Average	59.37
	<60	106 (43.98%)
	≥60	135 (56.01%)
Gender	Male	119 (49.37%)
	Female	122 (50.62%)
Stroke	Yes	11 (4.56%)
	No	230 (95.43%)
Creatinine clearance	Normal (≥85)	93 (38.58%)
	Moderate impairment (50-85)	119 (49.37%)
	Severe impairment (<50)	28 (11.61%)
	Dialysis	1 (0.41%)
Carotid stenosis	≥50%	18 (7.46%)
	Absence or <50%	223 (92.53%)
Extracardiac arteriopathy	Yes	24 (9.95%)
	No	217 (90.04%)
Poor mobility	Yes	5 (2.07%)
	No	236 (97.92%)
Previous cardiac surgery	Yes	31 (12.86%)
	No	210 (87.13%)
COPD	Yes	75 (31.12%)
	No	166 (68.87%)
Critical perioperative state	Yes	5 (2.07%)
	No	236 (97.92%)
Diabetes mellitus	Yes	38 (14.93%)
	No	203 (85.06%)
Diabetes on insulin	Yes	18 (7.46%)
	No	223 (92.53%)
Functional class (NYHA)	1	34 (14.10%)
	2	25 (10.37%)
	3	146 (60.58%)
	4	36 (14.93%)
Left ventricular function	Good (≥50%)	150 (62.24%)
	Moderate (30-50%)	65 (26.97%)
	Poor (<30%)	26 (10.78%)
Recent MI	Yes	4 (1.65%)
	No	237 (98.34%)
Pulmonary hypertension	Absent: SPAP <31 mmHg	169 (70.12%)
	Moderate: SPAP 31-55 mmHg	37 (15.35%)
	Severe: SPAP >55 mmHg	35 (14.52%)
Urgency	Elective	222 (92.11%)
	Urgency	15 (6.22%)
	Emergency	4 (1.65%)
Type of surgery	MV replacement	64 (26.55%)
	AV replacement	109 (45.22%)
	MV + AV surgery	19 (7.88%)
	MV repair	30 (12.44%)
	Isolated TV surgery	5 (2.07%)
	ASD or VSD correction	14 (5.8%)
EuroSCORE II	Average	3.97
	<2	119 (49.37%)
	≥2 to <4	64 (26.55%)
	≥4 to ≤6	20 (8.29%)
	>6	38 (15.76%)

ASD=atrial septal defect; AV=aortic valve; COPD=chronic obstructive pulmonary disease; MI=myocardial infarction; MV=mitral valve; NYHA=New York Heart Association; SPAP=systolic pulmonary artery pressure; TV=tricuspid valve; VSD=ventricular septal defect

**Table 2:** Risk factors for stroke.

		Stroke group (n=11) n (%)	Control group 1 (n=230) n (%)	*P* (Fisher's exact test)	OR (IC 95%)	*P*
Age	Average	61.19	57.60	0.05		
	<60	3 (27.2%)	103 (44.8%)			
	≥60	8 (72.7%)	127 (55.2%)	0.3554	2.1627 (0.5595-8.3604)	0.2635
Gender	Male	6 (54.5%)	113 (49.1%)			
	Female	5 (45.5%)	117 (50.9%)	0.7667	1.2425 (0.3688-4.1861)	0.7261
Creatinine clearance	Normal (≥85 mL/min)	4 (36.4%)	89 (38.7%)	1.0000	1.1046 (0.3143-3.8819)	0.8767
	Impaired (<85 mL/min)	7 (63.6%)	141 (61.3%)			
Carotid stenosis	≥50% lesion	3 (27.3%)	15 (6.5%)			
	Absence or <50% lesion	8 (72.7%)	215 (93.5%)	0.0396	5.3750 (1.2909-22.3805)	0.0208
Extracardiac arteriopathy	Yes	2 (18.2%)	22 (9.6%)			
	No	9 (81.8%)	208 (90.4%)	0.3007	2.1010 (0.4268-10.3438)	0.3613
Poor mobility	Yes	1 (9.1%)	4 (1.8%)			
	No	10 (90.9%)	226 (98.2%)	0.2099	5.6500 (0.5773-55.2959)	0.1368
Previous cardiac surgery	Yes	3 (27.3%)	28 (12.2%)			
	No	8 (72.7%)	202 (87.8%)	0.1552	2.7054 (10.8025-0.6775)	0.1589
COPD	Yes	4 (36.4%)	71 (30.9%)			
	No	7 (63.6%)	159 (69.1%)	0.7429	1.2797 (0.3630-4.5112)	0.7013
Critical perioperative state	Yes	1 (9.1%)	4 (1.8%)			
	No	10 (90.9%)	226 (98.2%)	0.2099	5.6500 (0.5773-55.2959)	0.1368
Diabetes mellitus	Yes	3 (27.3%)	35 (15.2%)			
	No	8 (72.7%)	195 (84.8%)	0.3868	2.0893 (0.5283-8.2620)	0.2935
Diabetes on insulin	Yes	1 (9.1%)	17 (7.4%)			
	No	10 (90.9%)	213 (92.6%)	0.5822	1.2529 (0.1513-10.3779)	0.8344
NYHA	I and II	4 (36.4%)	55 (24.0%)	0.4707	0.5500 (0.1552-1.9493)	0.3544
	III and IV	7 (63.6%)	175 (76.0%)			
Left ventricular function	Normal (≥50%)	8 (72.7%)	142 (61.7%)			
	Impaired (<50%)	3 (27.3%)	88 (38.3%)	0.5416	0.6051 (0.1564-2.3419)	0.4669
Recent MI	Yes	1 (9.1%)	3 (1.3%)			
	No	10 (90.9%)	227 (98.7%)	0.1714	7.5667 (0.7215-79.3529)	0.0915
Pulmonary hypertension (SPAP >30 mmHg)	Yes	7 (63.6%)	162 (70.4%)			
	No	4 (36.4%)	68 (29.6%)	0.7372	1.3613 (0.3859-4.8028)	0.6315
Urgency	Elective	10 (90.9%)	212 (92.2%)	0.6028	0.8491 (0.1028-7.0119)	0.8793
	Non-elective	1 (9.1%)	18 (7.8%)			
Type of surgery	MV replacement	3 (27.3%)	61 (26.5%)			
	AV replacement	7 (63.6%)	102 (44.3%)			
	MV + AV surgery	0	19 (8.2%)			
	MV replacement	1 (9.1%)	29 (12.6%)			
	TV surgery	0	5 (2.2%)			
	ASD or VSD	0	14 (6.1%)	0.7327		
EuroSCORE II	Average	5.38±7.79	3.98±7.14	0.5361		
	<2	6 (54.5%)	113 (49.1%)			
	2 to 4	1 (9.1%)	63 (27.3%)			
	4 to 6	1 (9.1%)	19 (8.3%)			
	>6	3 (27.3%)	35 (15.3%)	0.5006		
CPB time**	Average	106.4	112.3	0.8		

ASD=atrial septal defect; AV=aortic valve; COPD=chronic obstructive pulmonary disease; CPB: cardiopulmonary bypass; MI=myocardial infarction; MV=mitral valve; NYHA=New York Heart Association; SPAP=systolic pulmonary artery pressure; TV=tricuspid valve; VSD=ventricular septal defect **CPB time: 162 patients, some did not show CPB time.

**Table 3:** Risk factors for carotid stenosis.

		Carotid stenosis ≥50% (n=18) n (%)	Control group 2 (n=223) n (%)	*P* (Fisher's exact test)	OR (IC 95%)	*P*
Age	Average	61.25	59.88	0.9		
	<60	4 (22.2%)	102 (45.7%)			
	≥60	14 (77.8%)	121 (54.3%)	0.0817	2.9504 (0.9417-9.2439)	0.0633
Gender	Male	9 (50.0%)	110 (49.3%)			
	Female	9 (50.0%)	113 (50.7%)	1.0000	1.0273 (0.3931-2.6843)	0.9562
Creatinine clearance	Normal (≥85 mL/min)	3 (16.6%)	90 (40.3%)			
	Impaired (<85 mL/min)	15 (83.4%)	133 (59.6%)	0.0752	3.3835 (0.9519-12.0257)	0.0596
Extracardiac arteriopathy	Yes	10 (55.6%)	14 (6.3%)			
	No	8 (44.4%)	209 (93.7%)	<0.0001	18.6607 (6.3644-54.7143	<0.0001
Poor mobility	Yes	1 (5.5%)	4 (1.8%)			
	No	17 (94.4%)	219 (98.2%)	0.3239	3.2206 (0.3407-30.4409)	0.3075
Previous cardiac surgery	Yes	2 (11.1%)	29 (13.0%)			
	No	16 (88.9%)	194 (87.0%)	1.0000	0.8362 (0.1827-3.8268)	0.8177
COPD	Yes	11 (61.1%)	64 (28.7%)			
	No	7 (38.9%)	159 (71.3%)	0.0071	3.9040 (1.4491-10.5179)	0.0071
Critical perioperative state	Yes	0	5 (2.2%)			
	No	18 (100%)	218 (97.8%)	1.0000	1.0737 (0.0571-20.1837)	0.9621
Diabetes mellitus	Yes	6 (33.3%)	32 (14.3%)			
	No	12 (66.6%)	191 (85.7%)	0.0450	2.9844 (1.0453-8.5204)	0.0411
Diabetes on insulin	Yes	2 (11.1%)	16 (7.2%)			
	No	16 (88.9%)	207 (92.8%)	0.6315	1.6172 (0.3414-7.6613)	0.5447
NYHA	I and II	1 (5.6%)	58 (26%)			
	II and IV	17 (94.4%)	165 (74%)	0.0824	5.9758 (0.7779-45.9065)	0.0857
Left ventricular function	Normal (≥50%)	10 (55.5%)	140 (62.8%)	0.6158	1.3494 (0.5123-3.5545)	0.5442
	Impaired (<50%)	8 (44.5%)	83 (37.2%)			
Recent MI	Yes	2 (11.1%)	2 (0.9%)			
	No	16 (88.9%)	221 (99.1%)	0.0289	13.8125 (1.8239-104.6052)	0.0110
Pulmonary hypertension (SPAP >30 mmHg)	Yes	14 (77.7%)	155 (69.5%)			
	No	4 (22.3)	68 (30.5%)	0.5967	0.6513 (0.2068-2.0511)	0.4638
Urgency	Elective	17 (94.4%)	205 (91.9%)	1.0000	0.6699 (0.084205.3282)	0.7050
	Non-elective	1 (5.5%)	18 (8.1%)			
Type of surgery	MV replacement	3 (16.6%)	61 (27.3%)			
	AV replacement	10 (55.5%)	99 (44.4%)			
	MV + AV surgery	3 (16.6%)	16 (7.2%)			
	MV replacement	1 (5.5%)	29 (13%)			
	TV surgery	1 (5.5%)	4 (1.8%)			
	ASD or VSD	0	6.3 (14%)	0.2978		
EuroSCORE II	Average	6.08	3.77	0.74		
	<2	3 (1.6%)	116 (52%)			
	≥2 to <4	5 (2.8%)	59 (26.4%)			
	≥4 to ≤6	4 (2.2%)	16 (7.2%)			
	>6	6 (1.8%)	32 (14.3%)	0.0056		

ASD=atrial septal defect; AV=aortic valve; COPD=chronic obstructive pulmonary disease; MI=myocardial infarction; MV=mitral valve; NYHA=New York Heart Association; SPAP=systolic pulmonary artery pressure; TV=tricuspid valve; VSD=ventricular septal defect

## DISCUSSION

While the vast majority of studies deal with the incidence of stroke in patients undergoing coronary artery bypass graft surgery, few are concerned with evaluating the incidence of stroke in other populations, such as those submitted to non-coronary surgery, such as valve and congenital surgeries in adults. This article is the first in a comprehensive review of the literature addressing the relationship between carotid stenosis, stroke and non-coronary cardiac surgery.

In Brazilian services, the number of degenerative valve diseases has been increasing, in addition to a high prevalence of rheumatic diseases, resulting in a large number of valve surgeries in the country. Therefore, an investigation of the predisposing causes of stroke in this population is justified^[20]^. Studies performed with students in some Brazilian capitals have estimated the prevalence of chronic rheumatic heart disease in 1-7 cases/1,000 students, which is significantly higher than the prevalence of the disease in developed countries, such as the United States, where it varies between 0.1 and 0.4 cases/1,000 students^[20]^.

Stroke is a complication that occurs in 2% of cardiac surgeries. It remains one of the most important causes of mortality and morbidity and is responsible for a great increase in health expenses^[2,3^. The incidence of 4.56% neurological ischemic events in this article is similar to that described in 2001 by Bucerius et al., who observed a rate of 4.6% of neurological injury after cardiac surgery^[21]^. However, it is important to emphasize the difference between the populations addressed by this study and by Bucerius et al.^[21]^ In this study, we considered only the ischemic events with stroke documented by CT image and compatible clinical condition in patients over 40 years old and non-coronary surgery, whereas that study includes strokes and TIAs in all types of surgery at different ages.

In 2012, Roffi et al.^[3]^ observed that in coronary surgeries stroke occurs in the first hours of the perioperative period (from anesthesia to anesthetic recovery) in 45% of cases and on the second day onwards in 55% of the cases. Early embolism might occur due to manipulation of the heart and aorta or particles carried by CPB; late events may be related to atrial fibrillation, myocardial infarction, low output, and hypercoagulability^[3]^.

Patients with carotid stenosis have an increased incidence of ischemic stroke, however, embolism remains the leading cause of ischemic events, 62% according to Selim, even among patients with carotid disease^[9]^. In this sample, the presence of CS ≥50% was the only risk factor associated with stroke, 27.3% of the patients with CS ≥50% developed ischemic stroke at some time in the perioperative period, demonstrating a strong association between stenosis and neurological damage.

In a study involving risk of stroke in coronary and non-coronary surgery, Costa et al.^[22]^ showed that carotid stenosis ≥70% was found in 3.47% of the patients and ≥50% in 9.63% of the patients studied. Patients without carotid stenosis or with lesions <50% presented 3.6% of stroke, while patients with lesions ≥ 50% and ≥70% showed 10 and 20% of strokes, respectively. The risk factors for stroke were CS ≥70%, peripheral artery disease and the presence of diabetes in insulin use^[22]^.

It is commonly accepted that the progression of carotid stenosis is related to atherosclerotic plaque instability, and, consequently, increased risk of stroke^[1]^. Sabatei et al. found that the progression of carotid stenosis was associated with a 2.5% to 5% increase in the risk of developing stroke in the first three years^[6]^, while Kakkos et al.^[7]^ demonstrated that the risk of ipsilateral stroke over the next eight years in asymptomatic patients with plaque progression was 16%. Hirt described in an article the relationship between CS and the occurrence of an ipsilateral neurological event, and the annual advance of stenosis was 5.2% per year for the entire cohort. The progression of stenosis was associated with a five-fold increase in ipsilateral stroke risk for the following year, especially for categories above 50% of obstruction^[23]^. Naylor and Bown^[2]^, in a complex and extensive meta-analysis, demonstrated that symptomatic or non-symptomatic patients with CS ≥50% presented a 7.4% risk of stroke, and this risk increased to 9.1% with lesions greater than 80%.

Regarding patients with previous stroke, when TIA or carotid occlusion were excluded, the risk fell to 3.8% in a lesion of 50 to 99% and 2% in a lesion of 70 to 99%. The incidence of ipsilateral stroke in a patient with asymptomatic unilateral lesion between 50 and 99% was 2% and the risk of any stroke was 2.9%. This risk did not increase with the severity of stenosis from 70 to 99% × 80 to 99%. Patients with asymptomatic bilateral CS with lesions between 50 and 99% or lesions of 50 to 99% with contralateral occlusion had a 6.5% risk of stroke after cardiac surgery^[2]^. In this study, the surgical subtype that presented the highest incidence of stroke was aortic valve replacement, however, no statistical difference was observed, and we did not find data in the literature that could be compared with these results.

In our hospital a line of research in cardiac surgery and cerebrovascular disease has been implemented, and the use of Doppler in all patients over 40 years submitted to cardiac surgery is part of the routine. However, outside the research environment, it is necessary to evaluate the cost-effectiveness of carrying out this procedure routinely. The detection of carotid disease is known to be a risk marker and may also be a cause of stroke. The use of carotid Doppler in the preoperative period of patients undergoing coronary surgery is well accepted, however there are no studies evaluating its use in non-coronary heart surgery. The indiscriminate use of carotid Doppler could only add cost without significant benefit. This series used the carotid Doppler routinely in all patients over 40 years old and showed carotid stenosis in 7.46% of the cases, requiring 13 examinations to detect a patient with the alteration. However, when assessing risk factors, the risk of carotid lesion was seen to increase significantly in the presence of diabetes mellitus, COPD, recent MI and extracardiac arteriopathy, which are markers of atherosclerosis as a whole. This seems to point to the need to routinely perform carotid Doppler in the presence of any of these risk factors.

It is worth mentioning that renal dysfunction showed a trend of significance with *P*=0.05. In a sample enlargement it might also become a risk marker. The increased EuroSCORE II was also associated with a higher incidence of carotid injury, noting that CS alone increases the EuroSCORE II value. Interestingly, there was no statistical difference between the mean age of patients with or without CS ≥50% (61.25 and 59.88 years, respectively), which can be attributed to the small sample size.

The relationship between CS and stroke is undeniable considering the available evidence, but it is very controversial whether plaque is the cause or a risk marker for stroke, since patients with carotid atherosclerotic disease may have severe atherosclerotic disease of the ascending aorta, which is an important cause of embolic stroke^[24]^. Subgroup analysis of the European Carotid Surgery Trial (ECST) and the North American Symptomatic Carotid Endarterectomy Trial (NASCET) have shown that the severity of stenosis (but non-occlusion) is a predictive risk factor for stroke in patients undergoing surgical treatment^[25,26]^. The results found in our study suggest that carotid lesions are associated to stroke. Nevertheless, they could not be confirmed as its etiology. The number of patients with carotid injury and stroke was small, only three cases. Although stroke was ipsilateral to the carotid lesion in these three patients, this does not establish a causal relationship.

The importance of aortic manipulation as a cause of stroke is known; plaques can rupture during cannulation and aortic clamping. Since open valve surgeries depend on CPB, these procedures cannot be avoided. Special care in handling the aorta mainly in the presence of calcification may minimize embolization, although many times the plaques may be soft and undetectable by the surgeon during the procedure. Strategies to avoid aortic manipulation in coronary surgeries are gaining ground with increased surgeon concern about the stroke outcome and its serious consequences. These strategies include non-CPB surgery and proximal anastomosis of internal thoracic artery grafts, avoiding periods of low output, treatment of arrhythmias, less use of CPB, avoiding transoperative hyperglycemia, improving cerebral oxygenation, endarterectomy and prior angioplasty in patients with severe carotid stenosis^[2,27,28]^.

In valve surgery, in addition to avoiding clamping and cannulation in regions of plaques, surgeons should be careful with calcium fragments, being routine in this service to wash the left ventricular cavity with saline solution and carefully aspirate it to remove small pieces of calcium that may break off in the removal of calcified cusps. Placing gauze in the left ventricle before removal of calcified aortic valves can also prevent pieces of calcium from falling between muscle trabeculae without being visualized.

These precautions may be useful, but it is necessary to alert surgical teams more consistently to the need for strategies to reduce the incidence of stroke in non-coronary operations. Strategies that seek to determine patients at greatest risk for stroke can assist the surgeon in conducting the procedure. The search for atherosclerosis markers may select patients at increased risk of stroke and guide that conduct. In addition to plaques in the aorta and stenosis in the extracranial carotid arteries, other factors interfere with cerebral perfusion, such as intracranial atherosclerosis. Therefore, maintaining adequate blood pressure and oxygenation is essential to avoid prolonged shock or significant hypoxemia, which can lead to brain damage. In our service, it is common practice to maintain mean arterial pressure above 70 mmHg in patients at increased risk of stroke, especially in patients with an important carotid injury, in addition to careful choice of cannulation site and aortic clamping.

Prophylactic treatment of severe carotid lesions in patients without neurological symptoms undergoing cardiac surgery is still controversial. Touze et al.^[4]^ reported that patients over 75 years of age and with severe bilateral carotid stenosis undergoing prophylactic carotid artery endarterectomy, that is, before cardiac surgery, had a 12% incidence of stroke after cardiac procedure. In 2012, Barrera et al.^[5]^ investigated the incidence of stroke in the postoperative period of cardiac surgery in patients with carotid artery stenosis above 70% who underwent carotid artery stent implantation and found that the group under study had a 6% risk of stroke and death in the first 30 days. Revelo et al., in a Brazilian sample of 1,169 patients submitted to coronary surgery, showed that 19.9% presented CS ≥50%, 8.6% had CS ≥70% and 2% had unilateral occlusion. Among patients with severe CS who underwent intervention for preoperative treatment, the incidence of TIA, stroke and death was 12.5% *versus* 3.4% for patients not undergoing CS treatment (*P*=0.24)^[12]^. For Naylor and Bown, carotid lesions would be considered markers of stroke rather than associated with its etiology, since they observed that groups of patients with unilateral stenosis above 50% or above 70% had no change in the incidence of stroke^[2]^.

The guideline of the American Heart Association recommends that all patients with 70-99% of asymptomatic carotid stenosis should receive risk control and treatment adequacy. They recommend that highly selected patients should be treated prophylactically with carotid artery endarterectomy or carotid artery stent when the risk of the procedure is lower than 1%^[11]^. In order to identify the best practice in this group of patients, it is necessary to carry out randomized multicenter studies with high statistical power, comparing prophylactic strategies for endarterectomy and/or carotid angioplasty and absence of prophylactic treatment, and taking into account the degree of stenosis and the presence of unilateral or bilateral carotid^[3]^. In 2015, Kang published a study on the effects of smoking on arterial disease including 667 patients. Those over 65 years old and smokers showed greater rupture and inflammation in atherosclerotic plaques, regardless of the plaque site, 8.3% compared to 3.9% for nonsmokers (*P*=0.04)^[29]^. In the present sample, COPD can be an inflammatory marker, inferring that there is a systemic inflammatory state triggered by smoking, which is associated with a greater likelihood of presenting concomitant carotid lesion, as well as a greater chance of developing extracardiac arteriopathy, that is, in other places besides the carotid arteries. This paper presents some limitations that should be emphasized: it is a database review and not a prospective study, and it was conducted in a single center with a small sample. The small number of events decreases the statistical power of the sample. In our study, we had only 11 cases of stroke, which limited the performance of the multivariate analysis, and other larger studies are necessary for better clarification. Furthermore, we did not know the duration of surgeries or the incidence of atrial fibrillation in the perioperative period.

## CONCLUSION

In the result of this study the incidence of ischemic stroke was 4.56%, while the incidence of carotid stenosis ≥50% was 7.46%. The significant risk factor for perioperative stroke in cardiac surgery was carotid stenosis ≥50%, while the factors associated with stenosis ≥50% were extracardiac arteriopathy, recent myocardial infarction, diabetes, COPD and higher EuroSCORE II.

**Table:** 

Authors’ roles & responsibilities	
MACC	Substantial contributions to the conception or design of the work; or the acquisition, analysis, or interpretation of data for the work; drafting the work or revising it critically for important intellectual content; agreement to be accountable for all aspects of the work in ensuring that questions related to the accuracy or integrity of any part of the work are appropriately investigated and resolved; final approval of the version to be published
JPN	Substantial contributions to the conception or design of the work; or the acquisition, analysis, or interpretation of data for the work; final approval of the version to be published;
JMO	Substantial contributions to the conception or design of the work; or the acquisition, analysis, or interpretation of data for the work; final approval of the version to be published
ALB	Substantial contributions to the conception or design of the work; or the acquisition, analysis, or interpretation of data for the work; final approval of the version to be published
MDS	Agreement to be accountable for all aspects of the work in ensuring that questions related to the accuracy or integrity of any part of the work are appropriately investigated and resolved; final approval of the version to be published
RZG	Agreement to be accountable for all aspects of the work in ensuring that questions related to the accuracy or integrity of any part of the work are appropriately investigated and resolved; final approval of the version to be published
ESSR	Agreement to be accountable for all aspects of the work in ensuring that questions related to the accuracy or integrity of any part of the work are appropriately investigated and resolved; final approval of the version to be published

## References

[1] Naylor AR, Schoroeder TV, Sillesen H (2014). Clinical and imaging features associated with an increased risk
of late stroke in patients with asymptomatic carotid disease. Eur J Vasc Endovasc Surg.

[2] Naylor AR, Bown MJ (2011). Stroke after cardiac surgery and its association with
asymptomatic carotid disease an updated systematic review and
meta-analysis. Eur J Vasc Endovasc Surg.

[3] Roffi M, Ribichini F, Castriota F, Cremonesi A (2012). Management of combined severe carotid and coronary artery
disease. Curr Cardiol Rep.

[4] Touze E, Warlow CP, Rothwell PM (2006). Risk of coronary and other nonstroke vascular death in relation
to the presence and extent of atherosclerotic disease at the carotid
bifurcation. Stroke.

[5] Barrera JG, Kristin E, Rojas BA, Balestrini C, Espinel C, Figueredo A (2012). Early results after synchronous carotid stent placement and
coronary artery bypass graft in patients with asymptomatic carotid
stenosis. J Vasc Surg.

[6] Sabatei S, Schlager O, Exner M, Melkusch W, Amighi J, Dick P (2007). Progression of carotid stenosis detected by duplex
ultrasonography predicts adverse outcomes in cardiovascular high-risk
patients. Stroke.

[7] Kakkos SK, Nicolaides NA, Charalambous I, Thomas D, Giannopoulos A, Naylor AR (2014). Predictors and clinical significance of progression or regression
of asymptomatic carotid stenosis. J Vasc Surg.

[8] Inzitari D, Eliasziw M, Gates P, Sharpe BL, Chan RKT, Meldrum HE (2000). The causes and risk of stroke in patients with asymptomatic
internal-carotid-artery stenosis. N Engl J Med.

[9] Selim M (2007). Perioperative stroke. N Engl J Med.

[10] Mahmoudi M, Hill PC, Xue Z, Torguson R, Ali G, Boyce SW (2011). Patients with severe asymptomatic carotid artery stenosis do not
have a higher risk of stroke and mortality after coronary bypass
surgery. Stroke.

[11] Goldstein LB, Bushnell CD, Adams RJ, Appel LJ, Braun LT, Chatuverdi S (2011). Guidelines for the primary prevention of stroke a guideline for
healthcare professionals from the American Heart Association / American
Stroke Association. Stroke.

[12] Revelo MSC, Oliveira DP, Arantes FBB (2013). Influence of Carotid Injury in Post-Myocardial Revascularization
Surgery and Its Late Evolution. Arq Bras Cardiol.

[13] Udess R, Solanki P, Mehta A (2017). Carotid artery stenosis as an independent risk factor for
perioperative strokes following mitral valve surgical
intervention. J Neurol Sci.

[14] Filsoufi F, Rahmanian PB, Castilho JG et al.Incidence.imaging analysis.and early and late
outcomes of stroke after cardiac valve operation (2008). Am. J Cardiol.

[15] Al Mosa AF, Omair A, Arifi AA, Najm HK (2016). Mitral valve replacement for mitral stenosis a 15-years single
center experience. J Saudi Heart Assoc.

[16] Messé SR, Acker MA, Kasner SE (2014). Stroke after aortic valve surgery results from a prospective
cohort. Circulation.

[17] Nashef SA, Roques F, Sharples LD, Nilsson J, Smith C, Goldstone AR (2012). EuroSCORE II. Eur J Cardiothorac Surg.

[18] Hirotani T, Kameda T, Kumamoto T, Shirota S, Yamano M (2000). Stroke after coronary artery bypass grafting in patients with
cerebrovascular disease. Ann Thorac Surg.

[19] European Carotid Surgery Trialists&apos; Collaborative
Group (1998). Randomised trial of endarterectomy for recently symptomatic
carotid stenosis final results of the MRC European Carotid Surgery Trial
(ECST). Lancet.

[20] Meira ZM, Goulart EM, Colosimo EA, Mota CC (2005). Long term follow up of rheumatic fever and predictors of severe
rheumatic valvar disease in brazilian children and
adolescents. Heart.

[21] Bucerius J, Gummert JF, Borger MA, Walther T, Doll N, Onnasch JF (2003). Stroke after cardiac surgery a risk factor analysis of 16,184
consecutive adult patients. Ann Thorac Surg.

[22] Costa MAC, Gauer MF, Gomes RZ, Schafranski MD (2015). Risk factors for perioperative ischemic stroke in cardiac
surgery. Braz J Cir Cardiovasc.

[23] Hirt LS (2014). Progression rate and ipsilateral neurological events in
asymptomatic carotid stenosis. Stroke.

[24] Mickleborough LL, Walker PM, Takagi Y, Ohashi M, Ivanow J, Tamariz M (1996). Risk factors for stroke in patients undergoing coronary artery
bypass grafting. J Thorac Cardiovasc Surg.

[25] Naylor AR, Rothwell PM, Bell PRF (2003). Overview of the principal results and secondary analyses from the
European and North American randomized trials of endarterectomy for
symptomatic carotid stenosis. Eur J Vasc Endovasc Surg.

[26] Lobo Filho JG, Leitão MCA, Lobo Filho HG, Soares JPH, Magalhães GA, Leão Filho CSC (2002). Cirurgia de revascularização Coronariana esquerda
sem CEC e sem manuseio da aorta em pacientes acima de 75
anos. Rev Bras Cir Cardiovasc.

[27] Sá MPBO, Ferraz PE, Escobar RR, Martins WN, Lustosa PC, Nunes EO (2012). Off-pump versus on-pump coronary artery bypass surgery
meta-analysis and meta-regression of 13,524 patients from randomized
trials. Rev Bras Cir Cardiovasc.

[28] Sedrakyan A, Wu AW, Parashar A, Bass EB, Treasure T (2006). Off-pump surgery is associated with reduced occurrence of stroke
and other morbidity as compared with traditional coronary artery bypass
grafting a meta-analysis of systematically reviewed trials. Stroke.

[29] Kang SJ, Mintz GS, Weiz G, Mehran R, Rabbani LE, Verheye S (2015). Age-related effects of smoking on coronary artery disease
assessed by gray scale and virtual histology intravascular
ultrasound. Am J Cardiol.

